# Limitations of PHQ-9 as a first line mental health screener in primary care

**DOI:** 10.3389/fpsyt.2026.1823578

**Published:** 2026-10-06

**Authors:** Jennifer Jane Newson, Dhanya Parameshwaran, Jason Schweitzer, Tara C. Thiagarajan

**Affiliations:** 1 Sapien Labs, Arlington, VA, United States; 2 Sapien Labs Center for Human Brain and Mind at Krea, IFMR, Chennai, India; 3 Department of Psychiatry, University of California San Diego, La Jolla, La Jolla, CA, United States

**Keywords:** assessment, depression, mental health, MHQ, PHQ-9, primary care, screening, triage

## Abstract

**Background:**

The PHQ-9, originally designed as an assessment for depression, is now widely used as a first line screener in primary care and is often the sole mental health screener used. However, it is currently unknown how well the PHQ-9 performs as a general screener for mental distress. Here we aim to determine the sensitivity and specificity of the (PHQ-9) for capturing general or transdiagnostic mental distress, defined as experiencing 5 or more severe mental health symptoms.

**Methods:**

PHQ-9 responses were collected alongside responses to the transdiagnostic Mental Health Quotient (MHQ) assessment that captures 47 symptoms spanning 10 disorders as well as additional elements from RDoC in a sample of 5,750 respondents collected in 2022. PHQ-9 sum scores were compared with those with 5 or more symptoms from the MHQ.

**Results:**

At a sum score cut-off corresponding to moderately severe depression, PHQ-9 had low sensitivity, capturing only 46% of those with general mental distress. At a cut-off corresponding to mild depression, sensitivity improved to 93%, while specificity dropped to 57%. Concurrently, the PHQ-9 missed several symptoms that were highly prevalent in the sample population and individuals with *Severe* PHQ-9 sum scores showed substantial heterogeneity in their overall symptom profiles.

**Conclusions:**

Altogether, the PHQ-9, due to its origin as a depression screener, is not appropriate as a screener for general mental distress. In the context of primary care triage, this highlights the need for an alternative disorder agnostic first line screener that enables the identification of severe mental distress regardless of the specific profile of symptoms.

## Introduction

There has been a substantial increase in the prevalence and burden of mental health disorders, especially in youth, while suicide is the fourth leading cause of death among 15–29-year-olds (1–4). As a result, there is an increasingly unsustainable strain on existing mental health services, and a greater need for efficient triage and care pathways at the level of primary care. The American Psychiatric Association and the American Psychological Association support the use of the Patient Health Questionnaire (PHQ-9) as a screening measure for adult and adolescent depression, where it has good sensitivity and specificity for depression, as well as being easy to administer and free of cost (5–8). Consequently, the PHQ-9 is one of the most widely used instruments for depression screening across many different geographical populations and clinical contexts (8–15). The questionnaire consists of 9 items that match criteria for major depression as outlined in the Diagnostic and Statistical Manual of Mental Disorders, Fifth Edition (DSM-5) and respondents rate each item according to how frequently they have been bothered by the symptom over the last two weeks (5, 8, 9, 16). This provides clinicians with a broad-stroke evaluation in the context of major depression symptoms, including an indication of suicidal risk (17, 18). In the United States, the U.S. Preventive Services Task Force (USPSTF) has recommended regular screening for depression in primary care since 2002, whereas anxiety screening was only added in 2023, and no comparable mandate exists for either other mental health disorders or transdiagnostic mental health screening (19, 20). Following this CMS established a dedicated Medicare billing code and national coverage determination specifically for annual depression screening in 2011, that has no equivalent for broader or transdiagnostic mental health screening (21). As a result, while the PHQ-9 is sometimes used in conjunction with the GAD-7 or followed up with additional questions, it often functions as the only mental health measure captured in a primary care encounter (22). Given the rising rates of numerous other mental health challenges, an ideal triage system would capture all those with mental health distress irrespective of specific diagnosis and enable appropriate referral. As a depression-specific questionnaire, the PHQ-9 therefore risks missing at-risk individuals given that symptoms spanning multiple disorders is the norm rather than the exception (23–25). Here we examine how well the PHQ-9 operates as a first line screener of mental distress, defined as any mental health symptoms impacting the ability to navigate life’s challenges and function productively. We do so by comparing it against the Mental Health Quotient (MHQ), a transdiagnostic assessment that captures 47 individual symptoms across 10 common DSM-5 defined disorders (26–28).

## Materials and methods

### Data acquisition

Participants were recruited as part of the ongoing Global Mind Project through online advertisements placed on Meta and Google that targeted age-sex groups (18-85+) and geographical regions across broad based interests and key words (29). Participants were directed to the MHQ website (https://sapienlabs.org/mhq/) and took the assessment anonymously for the purpose of getting their mental health scores and personalized report. For this study, PHQ-9 questions were included along with the MHQ questions between August and September 2022 during which time, 6,005 respondents completed the English version of both the MHQ assessment as well as the PHQ-9 in sequence within the same online survey. A successful completion indicates that all items of both assessments were answered. Trends of marital status, educational attainment and mental health treatment status acquired by the American Community Survey and Household Pulse Surveys conducted by the United States Census Bureau are mirrored in the Global Mind Data for the United States (29). Nonresponse bias in other countries is currently unknown.

Only respondents who responded “Yes” to the MHQ question “Did you find this assessment easy to understand?” and completed the survey in a time frame appropriate for reading all questions (≥7 minutes) were included in the analysis, leading to a final sample size of 5,750. Respondents were aged 18-85, predominantly from 16 countries, with 42.6% of respondents reporting their biological sex as male (see **Supplementary Table 1**).

### The MHQ assessment

The MHQ captures 47 items including all symptoms across 10 major mental health disorders as well as items derived from Research Domain Criteria (RDoC) (26, 27). The MHQ items were obtained by coding the mental health functions and symptoms assessed in over 10,000 questions across 126 commonly used assessments spanning all major mental health disorders, transdiagnostic assessments as well as other frameworks in neuroscience and cognitive disorders (see (26) for more details and a full list of the 126 assessment tools). This resulted in the set of 47 unique categories of items which was the most parsimonious set of items that spanned all symptoms. The MHQ thus includes items that map to the PHQ-9 items (**Supplementary Table 2**). Within the MHQ, each of these 47 items were rated by respondents using a 1–9 life impact scale (i.e. a Likert scale with 9 positions) reflecting the impact on one’s ability to function (27). Of these 47 items, 27 were capacities (i.e. such as emotional control which could be both problematic or strengths and are therefore assessed on a bi-directional scale) and 20 were problems (i.e. such as suicidal thoughts which have no positive dimension and are assessed on a unipolar scale). Ratings from these 47 items are aggregated into a single score (the MHQ score) that positions individuals on a scale that is divided into six categories from *Distressed* to *Thriving* (30). The score is based on an algorithm that thresholds ratings as negative and positive based on the impact to function and applies a nonlinear transformation of the scale such that increasing negative impact to function is amplified (30). The resulting MHQ scores fall on a positive-negative continuum with the following criterion that have been validated in previous studies. The first criteria is to separate out those that would likely be associated with clinical diagnosis into the negative score range. In this regard we have previously shown that 89% of those with negative MHQ scores are associated with individuals having symptoms of functional impact mapping to one or more disorders (and the remaining 11% have typically 5+ symptoms that don’t align with any one specific disorder criteria but rather span multiple disorders) while <1% with positive scores map to any disorder criteria. The second criteria is, as a measure that broadly reflects the mental capacity to navigate life and function productively. In this regard the score has been shown to relate linearly to days per month unable to work (28). The score is thus an overall measure of mental health and wellbeing (in the positive direction) or conversely a measure of overall mental distress in the negative direction. Mental distress can thus be considered a unidimensional construct in that it reflects a mental challenge to the ability to navigate life and function productively. However, the specific mental aspects that are challenging across individuals may be heterogeneous.

The MHQ score has been shown to have strong sample-to-sample consistency as well as internal consistency using data from 179,298 people across eight English-speaking countries (28).

### PHQ-9 sum scores

Each PHQ-9 item was rated on a frequency scale of 0 to 3 that reflected how much a symptom had bothered them over the last two weeks (0=*Not at all*; 1=*Several days*; 2=*More than half the days*; 3=*Nearly every day*). The sum of these ratings, the PHQ-9 sum score, was computed for each respondent, and the proportion of respondents within each category (*None* = 0-4; *Mild* = 5-9; *Moderate* = 10-14; *Moderately severe* = 15-19; *Severe* **≥**20) was calculated.

### Analysis of PHQ-9 as a first line screener

While the PHQ-9 sum scores are typically divided into five categories (*None* to *Severe*), the MHQ scores are divided into six score categories (*Distressed* to *Thriving*). In order to determine the sensitivity and specificity of the PHQ-9 for identifying all those with mental distress, we calculated the percentage of respondents with ≥5 symptoms out of the 47 MHQ items, for each PHQ-9 and MHQ score category. In this context, an MHQ ‘symptom’ was an MHQ item that was rated ≥8 for those rated on a problem scale where 9 was the most severe, or ≤2 for those rated on a spectrum scale where 1 was the most negative impact.

We then compared the complete MHQ symptom profile for individuals with *Severe* PHQ-9 sum scores (scores ≥20; N = 373) and estimated the degree of difference between each pair of individuals by calculating the absolute difference in life impact ratings for each pair of respondents. To determine the heterogeneity of MHQ profiles for individuals with *Severe* PHQ-9 sum scores, we then computed the mean and standard deviation (SD) of these absolute differences for each pair of individuals for 12 MHQ items that matched to PHQ-9 items (see **Supplementary Table 2**) as well as the remaining 35 that did not match to PHQ-items. We then examined whether the distributions of the means and SDs for PHQ-9 matched and non-matched items were different by computing statistical significance with a two-sample t-test.

## Results

### Identification of those with transdiagnostic mental distress

In this general population sample, 20.2% had MHQ scores <0 (*Distressed* or *Struggling*), while 46.9% had MHQ scores between 101-200 (*Succeeding* or *Thriving*) (**Figure 1A**). For PHQ-9 sum scores, 16.2% had *Moderately severe* or *Severe* scores, while 42.0% had PHQ-9 sum scores of 0-4 (None), (**Figure 1B**). Overall, 30.6% had PHQ-9 sum scores ≥10, considered to indicate moderate to severe depression (5). We then asked what fraction of the population with any symptoms of mental distress not specifically aligned with depression were captured by the PHQ-9. The MHQ metric has been developed such that those with severe symptoms would be largely captured in the *Distressed/Struggling* categories, irrespective of the specific symptoms or disorder classification (28, 30). **Figure 1C** shows the percentage of those *within* each MHQ category that were in each PHQ-9 sum score group. While 55% of those in the MHQ *Distressed* category had a severe PHQ-9 sum score, 22% of had PHQ-9 sum scores in the *Moderate* to *None* ranges indicating individuals largely with symptoms outside of the depression framework. Conversely (not shown in figure), 84% of those with severe PHQ-9 scores were in the *Distressed* or *Struggling* MHQ categories and <5% were in the *Managing* or higher categories.

**Figure 1:**
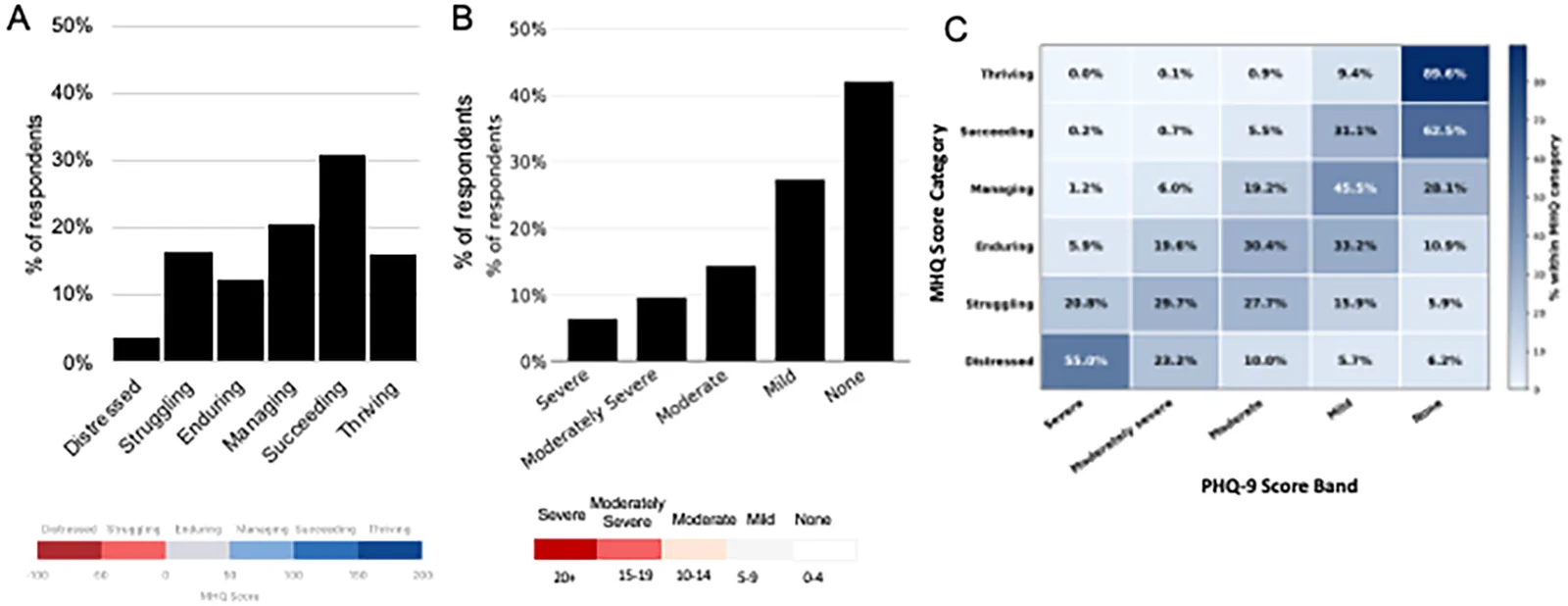
Distribution of PHQ-9 and MHQ scores within the sample. **(A)** Prevalence of respondents for each MHQ score category. **(B)** Prevalence of respondents for each PHQ-9 sum score category. **(C)** Co-occurrence matrix showing percentage of PHQ-9 sum score prevalence in each MHQ category.

The MHQ metric has been developed such that those with severe symptoms would be largely captured in the *Distressed/Struggling* categories, irrespective of the specific symptoms or disorder classification (28, 30). At the symptom level, the DSM criteria for diagnosis of mental health disorders typically involves the presence of five or more symptoms associated with a particular disorder definition. In this sample, 63.4% of those with ≥5 severe symptoms fell in the *Distressed* and *Struggling* categories while <1% fell in the *Succeeding* and *Thriving* categories (**Table 1**, left two columns). In contrast, those with ≥5 severe symptoms were spread almost evenly across the PHQ-9 score categories, with 20.3% to 26.5% in each of the PHQ-9 categories from *Mild* to *Severe*, while 7.3% were in the *None* category (**Table 1**, right 2 columns). Thus, if *Moderately severe* and above were used as the cut-off for triage, this would capture only 45.9% of those with ≥5 severe symptoms, representing a poor sensitivity. Conversely, if *Mild* and above were used, it would capture 92.7% of those with ≥5 severe symptoms but would have a high false positive rate such that 57%, or the majority of those captured by the screener, would not actually have mental distress.

**Table 1:** The percentage of individuals with ≥5 severe symptoms (as defined by their MHQ profile) for each of the MHQ and PHQ-9 score groups.

MHQ score group	Percentage distribution of people with ≤2 rating (spectrum item) or ≥8 (problem items) ratings for ≥5 items	PHQ-9 sum score group	Percentage distribution of people with ≤2 rating (spectrum item) or ≥8 (problem items) ratings for ≥5 items
Distressed	12.3%	Severe	19.7%
Struggling	51.1%	Moderately severe	26.2%
Enduring	23.0%	Moderate	26.5%
Managing	12.7%	Mild	20.3%
Succeeding	0.9%	None	7.3%
Thriving	0.0%		

### Heterogeneity of symptom profiles corresponding to “severe” PHQ-9

We next examined the answer ratings across the 47 MHQ and nine PHQ-9 items to understand why the PHQ-9 had poor specificity and sensitivity. To do so, we focused on the prevalence of symptoms (as identified in the MHQ) among respondents with a *Severe* PHQ-9 (i.e. sum score ≥20). Within the capacity items (those assessed on negative-positive scale), several items not assessed by PHQ-9, including ‘outlook and optimism’ (56.0%), ‘self-image’ (52.0%) and ‘physical intimacy’ (50.4%) ranked at similarly high prevalence levels to items within the PHQ-9 (e.g., ‘sleep quality’, 59.5%; ‘energy level’, 52.5%; ‘appetite regulation’, 47.5%) (**Figure 2A**). Similarly, among problem items (those assessed on a scale of increasing impact of the problem), ‘avoidance & withdrawal’ (65.7%), ‘fear & anxiety’ (63.5%), ‘unwanted, strange or obsessive thoughts’ (58.2%) ranked at similarly high levels of prevalence to ‘feelings of sadness, distress or hopelessness’ (76.1%) and ‘guilt & blame’ (61.9%) (**Figure 2B**). More importantly, this demonstrates that numerous symptoms outside of the depression criteria (**Figure 2**, grey bars) have equal or higher prevalence even relative to some of the depression symptoms (**Figure 2**, black bars) that are missed by the PHQ-9. Furthermore, analysis across the entire sample showed that symptom ranking was similar (**Supplementary Figure 1**) indicating that non depression symptoms have similarly high prevalence. This shows both that those individuals flagged by the PHQ-9 have highly heterogeneous symptom profiles and that a large fraction of people with non-depressive mental health symptoms are likely to be missed.

**Figure 2:**
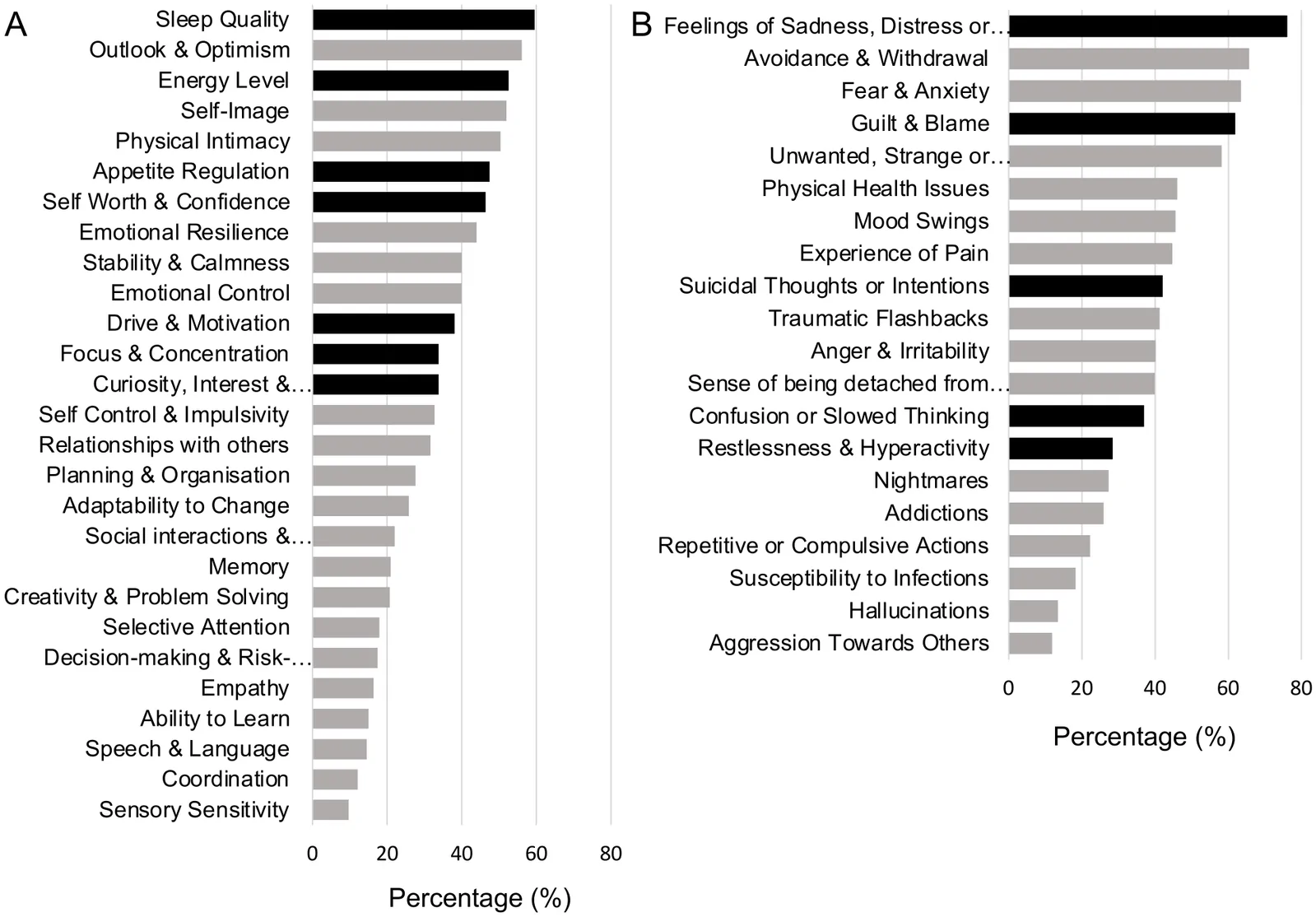
Prevalence of severe negative life impact ratings across 47 MHQ items for respondents with “severe” PHQ-9 sum scores. **(A)** The proportion of >7 ratings for respondents with “severe” PHQ-9 sum scores for each of 20 MHQ problem items. **(B)** The proportion of <3 ratings for respondents with “severe” PHQ-9 sum scores for each of the 27 spectrum items. MHQ items not assessed by the PHQ-9 shown in grey; 12 items that mapped to PHQ-9 item shown in black.

Given the diversity of symptomatic experience, even within the same disorder label (31, 32), we next explored the heterogeneity of MHQ symptom profiles for individuals with *Severe* PHQ-9 scores across the 12 MHQ items that mapped to the 9 items in the PHQ-9, as well as the additional 35 MHQ items (**Supplementary Table 2**; **Figures 3A, B**). For MHQ items not mapped to PHQ-9, the average difference in ratings of those items between pairs of respondents and corresponding standard deviation was 2.6 ± 3.1. In comparison, for the MHQ items that mapped to PHQ-9 it was 2.3 ± 2.8. While the MHQ rating differences between these two groups were statistically significant (*p* < 0.001 by t-test), the effect size was small (Cohen’s d=0.1) indicating that while PHQ-9 matched items had lower mean differences in life impact than those items not within PHQ-9, there was considerable variability within both groups. Coefficient of variation (CV) was 122% in percentage terms for PHQ-9 matched items and 119% for non-matched items. Ratings varied as much as 5 to 6 points on the 9-point life impact scale for individual items. **Figure 3C** shows illustrative examples of this heterogeneity. Finally, ratings of all items (depression related and non-depression related) differed between those with *Severe* PHQ-9 scores and those below the severe threshold (**Supplementary Table 3**) even if the rating was not in the symptomatic range reflecting the complex multi-disorder nature of symptom profiles. Overall, individuals within the *Severe* PHQ-9 group showed considerable variability in their broader symptom profile, and commonly experienced functionally impacting symptoms not assessed by PHQ-9.

**Figure 3:**
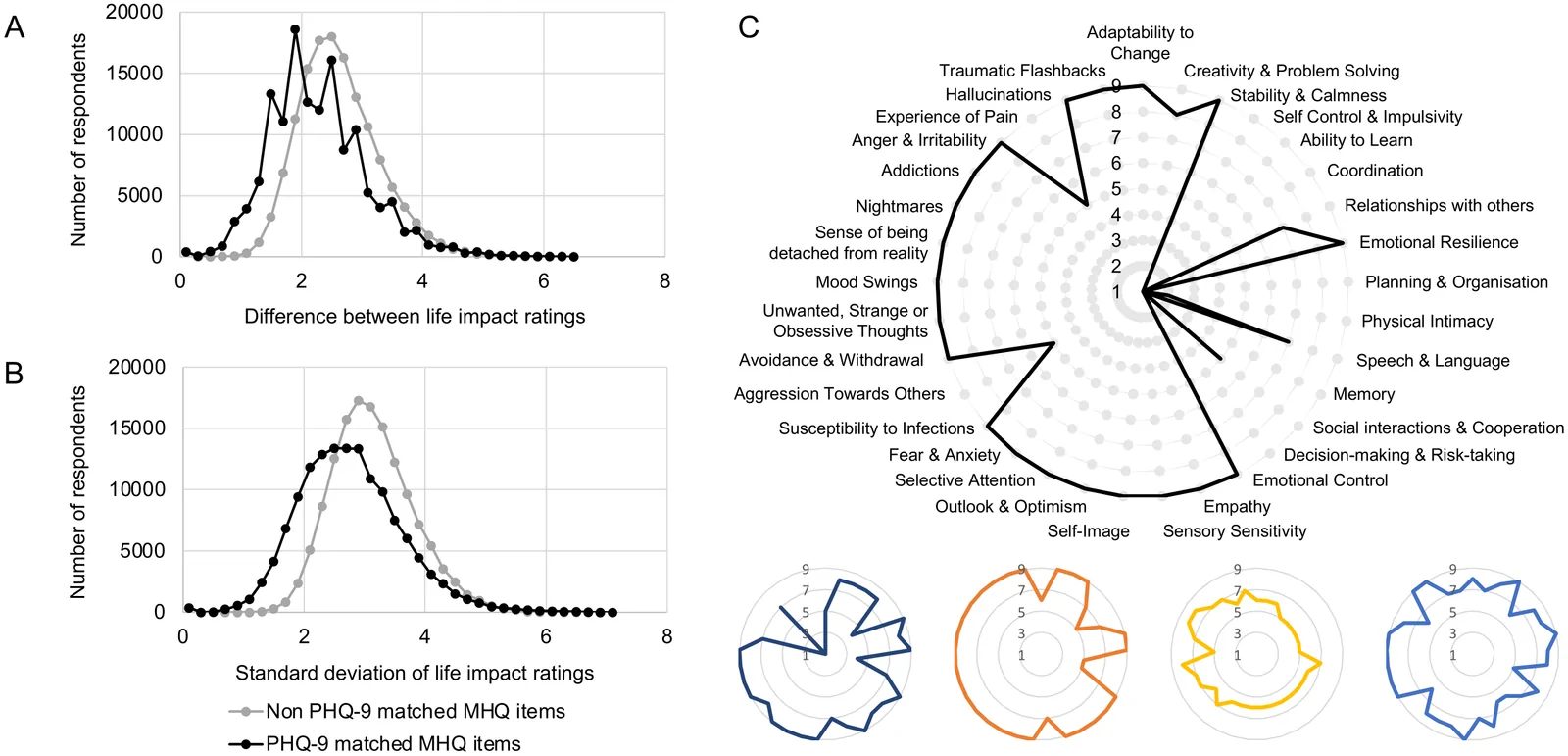
Variability of MHQ profiles for respondents with Severe PHQ-9 sum scores. **(A)** Distribution of the average absolute difference in life impact rating between all pairs of respondents with Severe PHQ-9 scores for the MHQ-mapped items **(black)** and MHQ items not in the PHQ-9 (grey). **(B)** Distribution of standard deviation of life impact rating between all pairs of respondents with Severe PHQ-9 scores for the MHQ-mapped items (black) and MHQ items not in the PHQ-9 (grey). **(C)** Example MHQ profiles of items not assessed by the PHQ-9 from 5 respondents with a Severe PHQ-9 sum score of 27. Each plot shows ratings of one respondent, larger plot shows labels for reference. Scale for spectrum items has been reversed so that higher values indicate more negative life impact in alignment with the problem scale.

## Discussion

The PHQ-9 has been shown to have high sensitivity and specificity for the criteria laid out by the DSM-5 for depression (5, 8, 9, 16), and is not meant to be a general screener for mental health problems. However, given that it is often the only first line mental health screener used in primary care, here we have evaluated how well it performs in this context. We show that it has poor sensitivity and specificity as a first line screener of overall mental distress. In particular, while the MHQ was able to more specifically identify individuals with ≥5 symptoms of any type, PHQ-9, as a depression screener, performed poorly in this transdiagnostic context. Rather, individuals with ≥5 severe symptoms were distributed across the categories from *Mild* to *Severe* with 7.3% even falling within the *None* category. Thus, if only those with *Severe* PHQ-9 scores were triaged to mental health care, this would miss almost 80% of those with mental health problems, representing a very low sensitivity. Conversely, if all those with *Mild* or higher PHQ-9 scores were considered, this would result in almost 80% of all individuals being triaged to mental health care despite only 20% actually having mental health problems, which is a very low specificity. This arises because the construct of depression does not encompass the full spectrum of symptoms that commonly arise alongside depression symptoms. For example, several symptoms that are among the most frequent in the population, even outside of anxiety, were not captured by the PHQ-9. The pattern where several symptoms unrelated to depression criteria arise more frequently than several depression symptoms themselves highlights the heterogeneity of depressive profiles. This reflects the challenge that a depression diagnosis can arise from 128 symptom combinations as per the DSM criteria, and that depression symptoms do not specifically cluster together among the larger set of symptoms (31, 32). This adds to previous evidence that PHQ-9, due to its purpose as a depression screener, misses the presence or intensity of symptoms outside these disorder criteria that are meaningful to patients (33, 34).

### The use of PHQ-9 as a first line screener

Screening for depression in primary care is useful, especially given rising rates of depression symptoms. The PHQ-9 as a short, easy to administer self-report instrument has been shown to have a sensitivity and specificity for clinical diagnosis of depression of 89% and 88% respectively (5, 8, 9, 16). However, while symptoms associated with depression have been rising, other symptoms and diagnoses have also rising in the population and are largely missed by the PHQ-9. With low sensitivity and specificity across the spectrum of symptom profiles, it has significant limitations as a stand-alone first-line screener and triage tool for general or transdiagnostic mental distress. While not the purpose for which it was not originally designed, it cautions the use of PHQ-9 as the starting point of evaluation by a mental health provider. While mental health providers may use the PHQ-9 along with other tools or clinical questioning, the identification of symptoms will be highly dependent on the clinician’s choice of additional assessments or questioning. It may also lead to patients being force fit into diagnoses of depression and/or anxiety as these are the tools used most frequently. This is a considerable challenge given the substantial heterogeneity of symptom profiles even among those with *Severe* PHQ-9 scores. Furthermore, this aligns with previous analysis showing that individuals with symptoms aligned to criteria of depression have overall symptom profiles that are almost as heterogeneous as symptom profiles between individuals with depression and other diagnoses such as attention-deficit/hyperactivity disorder (ADHD) (31). Thus, altogether, the PHQ-9 is not well positioned as a first line screener for general mental distress, and on its own risks force fitting individuals into a depression framework.

We note that the sample used in this study are self-selected respondents from a general population who are capable and interested in participating in an Internet-based self-assessment. It is therefore not a perfectly representative sample. In addition, the MHQ score has been evaluated for sensitivity and specificity for clinical diagnosis using mapping of self-report data to DSM diagnostic criteria rather than against clinician evaluations through interview and is therefore not an independent clinician-established diagnostic or transdiagnostic gold standard. In addition, the threshold of five or more severe MHQ symptoms is not a definitive reference standard for general mental distress. However, the high sensitivity and specificity of other self-report tools like the PHQ-9 to clinician evaluations suggest that self-report is a generally useful approach. Thus, it is unlikely that the overall outcomes of this study, which is that the PHQ-9 has poor sensitivity and specificity in a transdiagnostic context, would be changed by these limitations.

### Requirements of an ideal first line screener for primary care

The goal of a first line mental health screener in primary care should be to identify those in need of further mental health evaluation and support regardless of the specific profile of mental health symptoms. While numerous assessments exist for various disorder criteria, the substantial overlap of symptoms across disorders (23) and the lack of clear empirical clustering of symptoms into specific disorder groups (31, 32) call out the need for a disorder agnostic first line screener that considers the breadth of symptom experience in the population. With symptom profiles typically spanning multiple disorders a disorder-agnostic perspective can provide more effective triage and referrals. We note that the MHQ itself is not proposed as the preferred first-line screener in primary care but rather has been used as a comprehensive set of symptoms against which the PHQ-9 is evaluated. Rather, a first line screener must meet various practical criteria. Primary care appointments are typically time constrained. Any screener must therefore take less than 2–3 minutes to fit within the appointment workflow, and results must be easily and rapidly interpreted by the primary care physician to be useful in the appointment context. These criteria argue against the use of multiple screeners which would be time consuming and require substantial synthesis and interpretation by the provider. Rather a single short screener that takes into consideration the relative frequencies and distributions of symptoms in the population to provide an indication of whether or not further mental health evaluation and support is required would better serve the purpose. In addition, it may have to be adaptive in the questions it serves rather than being a static question set, as prevalence and profiles can differ across age groups and geographies. Finally, it would have to use more sophisticated algorithms than sum scores. One possibility is that it can utilize machine learning approaches to predict outcomes of a more comprehensive transdiagnostic assessment. Such as a screener would need to be constructed based on a comprehensive large-scale dataset of symptom profiles that are closely aligned with population prevalence.

## Conclusion

Overall, while PHQ-9 has high sensitivity and specificity for the criteria laid out by the DSM-5 for depression (5, 8, 9, 16), it has poor sensitivity and specificity as a first line transdiagnostic screener of mental distress in clinical practice. Altogether, over 50% of those with mental health struggles that do not fit with moderately severe depression will be missed, and this may contribute to the long struggle that patients experience to find effective treatment (35). This calls out the need for a rapid disorder agnostic first line screener that considers the breadth of symptom experience in the population rather than focusing on depression alone.

## Data Availability

The datasets presented in this study can be found in online repositories. The names of the repository/repositories and accession number(s) can be found below: https://sapienlabs.org/global-mind-project/researcher-hub/.
